# The complete chloroplast genome of *Papaver setigerum* and comparative analyses in Papaveraceae

**DOI:** 10.1590/1678-4685-GMB-2019-0272

**Published:** 2020-08-17

**Authors:** Luxian Liu, Yingxue Du, Cheng Shen, Rui Li, Joongku Lee, Pan Li

**Affiliations:** 1 Henan University, School of Life Sciences, Key Laboratory of Plant Stress Biology, Kaifeng, China.; 2 Zhejiang University, College of Life Sciences, The Key Laboratory of Conservation Biology for Endangered Wildlife of the Ministry of Education, Hangzhou, China.; 3 Food inspection and Testing Institute of Henan Province, Physical and Chemical Laboratory, Zhengzhou, China.; 4 Chungnam National University, Department of Environment and Forest Resources, Daejeon, South Korea.

**Keywords:** Papaver setigerum, chloroplast genomes, chloroplast hotspot, species identification

## Abstract

*Papaver setigerum* is an annual herb that is closely related to the opium poppy, *P. somniferum*. Genetic resources for *P. setigerum* are scarce. In the present study, we assembled the complete chloroplast (cp) genome of *P. setigerum* based on genome skimming data, and we conducted comparative cp genome analyses to study the evolutionary pattern in Papaveraceae. The cp genome of *P. setigerum* is 152,862 bp in length with a typical quadripartite structure. Comparative analyses revealed no gene rearrangement in the Papaveraceae family, although differences were evident in genome size, gene losses, as well as inverted repeats (IR) region expansion and contraction. The *rps*15 gene has been lost from the genomes of *Meconopsis racemosa*, *Coreanomecon hylomeconoides*, *P. orientale*, *P. somniferum*, and *P. setigerum*, and the *ycf*15 gene is found only in *C. hylomeconoides*. Moreover, 13 cpDNA markers, including *psb*A-*trn*H, *rps*16-*trn*Q, *trn*S-*trn*G, *trn*C-*pet*N, *trn*E-*trn*T, *trn*L-*trn*F, *trn*F-*ndh*J, *pet*A-*psb*J, *ndh*F-*rpl*32, *rpl*32-*trn*L, *ccs*A-*ndh*D, *ndh*E-*ndh*G, and *rps*15-*ycf*1, were identified with relatively high levels of variation within *Papaver*, which will be useful for species identification in this genus. Among those markers, *psb*A-*trn*H is the best one to distinguish *P. somniferum* and *P. setigerum*.

## Introduction

*Papaver setigerum* DC., an annual herb of the poppy family (Kalis, 1979), occurs in the Mediterranean region, especially in southwestern Europe (Portugal, Spain, France, Italy, Greece) and North Africa (Pignatti, 1982). This plant is closely related to and sometimes treated as a variety or subspecies of opium poppy (*P. somniferum* L.) due to its similarity in flower-shape, color, fruit, and production of small amounts of morphine alkaloids (La Valva *et al.*, 1985; Osalou *et al.*, 2013). Of the 110 species of the genus *Papaver*, only *P. somniferum* and *P. setigerum* are controlled species in most countries (Choe *et al.*, 2012). However, the cytological evidence shows that *P. somniferum* is diploid (2n = 22), while *P. setigerum* is tetraploid (2n = 44) (Fulton, 1944; Choe *et al.*, 2012), indicating that *P. setigerum* is not likely the wild ancestral species of the cultivated *P. somniferum* (Farmilo *et al.*, 1953). For *Papaver* species, inter-specific identification based only on morphological characteristics is difficult because of the similarities in appearance mentioned above (Osalou *et al.*, 2013). Phytochemical methods (Zhang and Cheng, 2009; Osalou *et al.*, 2013) and various molecular markers (Fan *et al.*, 1987; Hosokawa *et al.*, 2004; Choe *et al.*, 2012; Zhang *et al.*, 2015) have been used to identify *Papaver* species in previous studies. However, current studies involving *P. setigerum* have mostly focused on its chemical composition, largely ignoring its genetic background.

Chloroplasts (cp), the photosynthetic organelles of most green plants, are known to be derived from cyanobacteria through endosymbiosis and co-evolution (Dagan *et al.*, 2012; Asaf *et al.*, 2017). In most angiosperms, cp genomes have a typically circular and quadripartite structure. The genome size is usually from 115 to 165 kb in length, consisting of two regions of inverted repeats (IRs), separated by a large single-copy (LSC) region and a small single-copy (SSC) region (Wicke *et al.*, 2011). Compared with nuclear and mitochondrial genomes, the cp genome is more conserved, not only in gene content and organization, but also in genome structure (Raubeson and Jansen, 2005). Due to its relatively conserved gene content and simple structure, small size, uniparental inheritance, and the fact that it is non-recombinant, the cp genome has been used as an ideal model for phylogenetic reconstruction (Liu *et al.*, 2017), evolutionary and comparative genomic studies (Liu *et al.*, 2018b), species identification (Thomson *et al.*, 2010; Greiner *et al.*, 2015) and markers development (Liu *et al.*, 2018a). Currently, the rapid development and improvement of next-generation sequencing technology have made the assembly of the cp genome cheaper and more efficient compared with traditional sequencing (Alkan *et al.*, 2011). In addition, the releases of many assembly processes or pipelines, such as SOAPdenovo2 (Luo *et al.*, 2012), CLC Genomics Workbench (CLC Inc., Rarhus, Denmark) and GetOrganelle (Jin *et al.*, 2018), have made cp genome reconstruction easier and much more effective.

In the present study, one *P. setigerum* individual was selected for genome skimming, and the complete chloroplast genome sequence was assembled and reported. We also compared the cp genomes among representatives of Papaveraceae and detected highly divergent regions of the cp genomes within the genus *Papaver*.

## Material and Methods

### Plant material, DNA extraction, and sequencing

We extracted whole-genomic DNA from silica-dried leaf tissue of one cultivated *P. setigerum* plant collected in Taizhou (Zhejiang, China), using modified CTAB reagent Plant DNAzol (Invitrogen, Shanghai, China) according to the manufacturer’s protocol. High quality DNA was sheared to yield fragments with length less than or equal to 800 bp. The quality of fragmentation was checked on an Agilent Bioanalyzer 2100 (Agilent Technologies). The 500 bp short-insert length paired-end library was prepared and sequenced by Beijing Genomics Institute (BGI, Wuhan, China). The library was run in one lane of an Illumina HiSeq X10 and obtained reads with length of 150 bp.

### Chloroplast genome assembly and annotation

The raw reads were first screened for Phred score < 30 to remove low-quality sequences. In order to ensure the accuracy of cp genome assembly, we employed two different methods to assemble the cp genome. In the first method, all the remaining reads were assembled into contigs implemented in the CLC genome workbench (CLC Inc., Rarhus, Denmark). The parameters set in CLC were as follows: 200 bp for minimum contig length, 3 for deletion and insertion costs, bubble size of 98, 0.9 for length fraction and similarity fraction, and 2 for mismatch cost. Then, the principal contigs representing the cp genome were separated from the total contigs using a BLAST (NCBI BLAST V2.2.31) search, with the cp genome of *P. somniferum* set as the reference. The representative cp contigs were oriented and ordered on the basis of the reference cp genome, and the complete chloroplast genome of *P. setigerum* was reconstructed by connecting overlapping terminal sequences. In the second method, the cp genome of *P. setigerum* was de novo assembled using the GetOrganelle pipeline (Jin *et al.*, 2018), with SPAdes 3.10.1 as assembler (Bankevich *et al.*, 2012).

Geneious R11 (https://www.geneious.com) was used to annotate the cp genome of *P. setigerum*, and putative starts, stops, and intron positions were identified on the basis of comparisons with homologous genes of the *P. somniferum* cp genome. The tRNA genes were verified with tRNAscan-SE v1.21 (Schattner *et al.*, 2005) with the default setting. We drew the circular chloroplast genome map of *P. setigerum* using the OrganellarGenomeDRAW program (OGDRAW, Lohse *et al.*, 2013).

### Comparative chloroplast genomic analyses

In order to study the sequence variation within Papaveraceae, we downloaded multiple publicly available cp genomes of the family from GenBank to compare the overall similarities, using *Leontice incerta* (Berberidaceae, MH940295) as the reference, according to the results of Kim and Kim (2016). The GenBank accession numbers for the Papaveraceae species are as follows (Table S1): *P. orientale* (NC_037832), *P. rhoeas* (NC_037831), *P. somniferum* (NC_029434), *Meconopsis racemosa* (NC_039625), *Coreanomecon hylomeconoides* (NC_031446), and *Macleaya microcarpa* (NC_039623). The sequence identities of the seven Papaveraceae cp genomes were implemented in the mVISTA program with LAGAN mode (Frazer *et al.*, 2004). The cp DNA rearrangement analyses of seven Papaveraceae cp genomes were based on Mauve Alignment (Darling *et al.*, 2004).

### Molecular markers development for *Papaver*

In order to screen variable characters within *Papaver*, multiple alignments of the four *Papaver* species cp genomes were carried out using MAFFT version 7.017 (Katoh and Standley, 2013). The nucleotide diversity (*Pi*) was determined by calculating the total number of mutations (*Eta*) and average number of nucleotide differences (*K*) using DnaSP v5.0 (Librado and Rozas, 2009).

### Phylogenetic inferences

The phylogenetic relationships of Papaveraceae were inferred using the whole chloroplast genome sequences of seven species; two species from Ranunculaceae (*Ranunculus macranthus*) and Berberidaceae (*Leontice incerta*) were chosen as the outgroups, according to the results of Kim and Kim (2016). The phylogeny inferences were conducted using Bayesian inference (BI) and maximum likelihood (ML) methods. ML analysis was performed with RAxML-HPC v8.1.11 on the CIPRES cluster (Miller *et al.*, 2010) with GTR + I + G set as the best-fit nucleotide substitution model. BI analysis was implemented in MrBayes v3.2.3 using the same substitution model mentioned above (Ronquist and Huelsenbeck, 2003).

## Results

### *P. setigerum* cp genome assembly, organization and gene content

**Figure 1:**
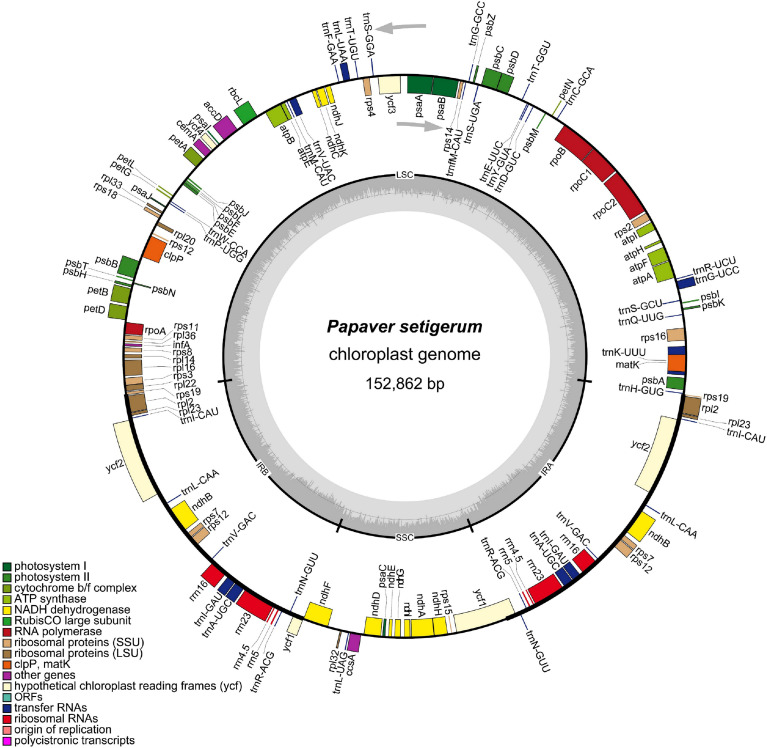
Chloroplast genome map of *Papaver setigerum*. Genes inside the circle are transcribed clockwise, genes outside are transcribed counter-clockwise. The light gray inner circle corresponds to the AT content, the dark gray to the GC content. Genes belonging to different functional groups are shown in different colors.

The complete cp genomes of *P. setigerum* assembled from two different assembly strategies were identical. However, using GetOrganelle to assemble the cp genome of *P. setigerum* was much faster and more effective than using CLC genome workbench (< 1h vs. > 6h). The cp genome size was 152,862 bp, and had a typical quadripartite structure that was similar to the majority of land plant cp genomes, consisting of an 83,022 bp large single copy region (LSC), a 17,944 bp small single copy region (SSC) and two 25,948 bp inverted repeats. The *P. setigerum* cp genome contains 113 unique genes, including 79 protein-coding genes, 30 tRNA genes and four ribosomal RNA genes (Figure 1 and Table 1). Eight protein-coding, seven tRNA, and four rRNA genes are duplicated and located in the IR regions. Among the 113 genes, nine protein-coding genes and six tRNA genes contain one intron; three protein-coding genes (*clp*P, *ycf*3 and *rps*12) contain two introns. We submitted the cp genome of *P. setigerum* to GenBank with the accession number MK820043.

**Table 1:** Genes contained in *P. setigerum* chloroplast genome (113 genes in total).

Category	Group of gene	Name of gene			
Self-replication	Ribosomal RNA genes	*rrn*4.5^a^	*rrn*5^a^	*rrn*16^a^	*rrn*23^a^
	Transfer RNA gene	*trnA*-UGC^a*^	*trnC-*GCA	*trnD-*GUC	*trnE-*UUC
		*trnF-*GAA	*trnfM*-CAU	*trnG-*GCC^*^	*trnG-*UCC
		*trnH-*GUG	*trnI-*CAU^a^	*trnI-*GAU^a*^	*trnK-*UUU^*^
		*trnL-*CAA^a^	*trnL-*UAA^*^	*trnL-*UAG	*trnM-*CAU
		*trnN-*GUU^a^	*trnP-*UGG	*trnQ-*UUG	*trnR-*ACG^a^
		*trnR-*UCU	*trnS-*GCU	*trnS-*GGA	*trnS-*UGA
		*trnT-*GGU	*trnT-*UGU	*trnV-*GAC^a^	*trnV-*UAC^*^
		*trnW-*CCA	*trnY-*GUA		
	Small subunit of ribosome	*rps*2	*rps*3	*rps*4	*rps*7^a^
		*rps8*	*rps*11	*rps*12^a,b**^	*rps*14
		*rps*15	*rps*16^*^	*rps*18	*rps*19^a^
	Large subunit of ribosome	*rpl*2^a*^	*rpl*14	*rpl*16^*^	*rpl*20
		*rpl*22	*rpl*23^a^	*rpl*32	*rpl*33
		*rpl*36			
	RNA polymerase subunits	*rpo*A	*rpo*B	*rpo*C1^*^	*rpo*C2
Photosynthesis	Subunits of photosystem I	*psa*A	*psa*B	*psa*C	*psa*I
		*psa*J	*ycf*3^**^		
	Subunits of photosystem II	*psb*A	*psb*B	*psb*C	*psb*D
		*psb*E	*psb*F	*psb*H	*psb*I
		*psb*J	*psb*K	*psb*L	*psb*M
		*psb*N	*psb*T	*psb*Z	
	Subunits of cytochrome	*pet*A	*pet*B^*^	*pet*D^*^	*pet*G
		*pet*L	*pet*N		
	Subunits of ATP synthase	*atp*A	*atp*B	*atp*E	*atp*F^*^
		*atp*H	*atp*I		
	Large subunit of Rubisco	*rbc*L			
	Subunits of NADH	*ndh*A^*^	*ndh*B^a*^	*ndh*C	*ndh*D
	Dehydrogenase	*ndh*E	*ndh*F	*ndh*G	*ndh*H
		*ndh*I	*ndh*J	*ndh*K	
Other genes	Translational initiation factor	*inf*A			
	Maturase	*mat*K			
	Envelope membrane protein	*cem*A			
	Subunit of acetyl-CoA	*acc*D			
	C-type cytochrome synthesis gene	*ccs*A			
	Protease	*clp*P^**^			
Unknown function	Conserved open reading frames	*ycf*1^a^(part)	*ycf*2^a^	*ycf*4	

a Two gene copies in IRs; ^b^ gene divided into two independent transcription units; one and two asterisks indicate one- and two-intron containing genes, respectively.

### Genome comparison of Papaveraceae

**Figure 2:**
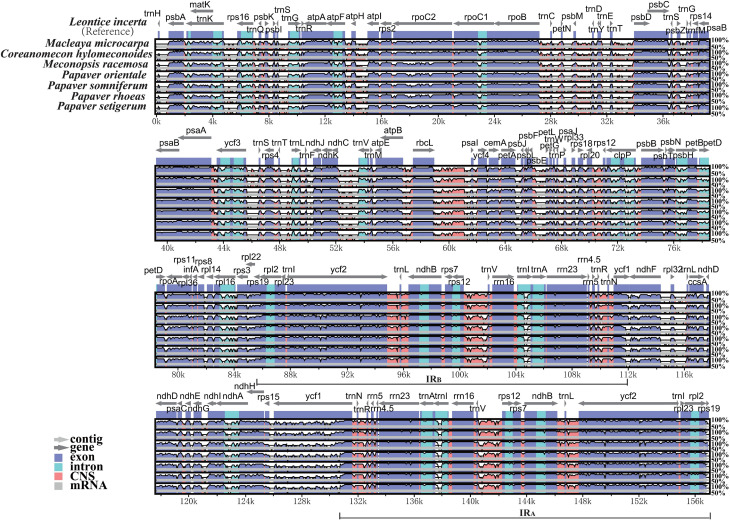
Visualization of alignment of the seven Papaveraceae chloroplast genome sequences, with *Leontice incerta* as the reference. The horizontal axis indicates the coordinates within the chloroplast genome. The vertical scale indicates the percentage of identity, ranging from 50 to 100%. Genome regions are color coded as protein coding, intron, mRNA, and conserved non-coding sequences (CNS).

**Figure 3:**
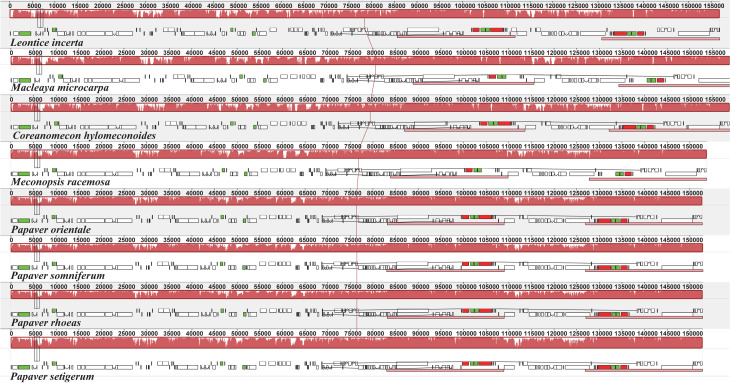
MAUVE alignment of seven Papaveraceae chloroplast genomes. *Leontice incerta* is shown at the top as the reference. Within each of the alignments, local collinear blocks are represented by blocks of the same color connected by lines.

The chloroplast genomes of the seven Papaveraceae species were relatively conservative, and the IR region is more conserved compared to the LSC and SSC regions (Figure 2). No rearrangements, such as translocations or inversions, occurred in gene organization after verification in this family (Figure 3). However, differences existed in genome size, gene losses, and IR expansion and contraction.

**Figure 4:**
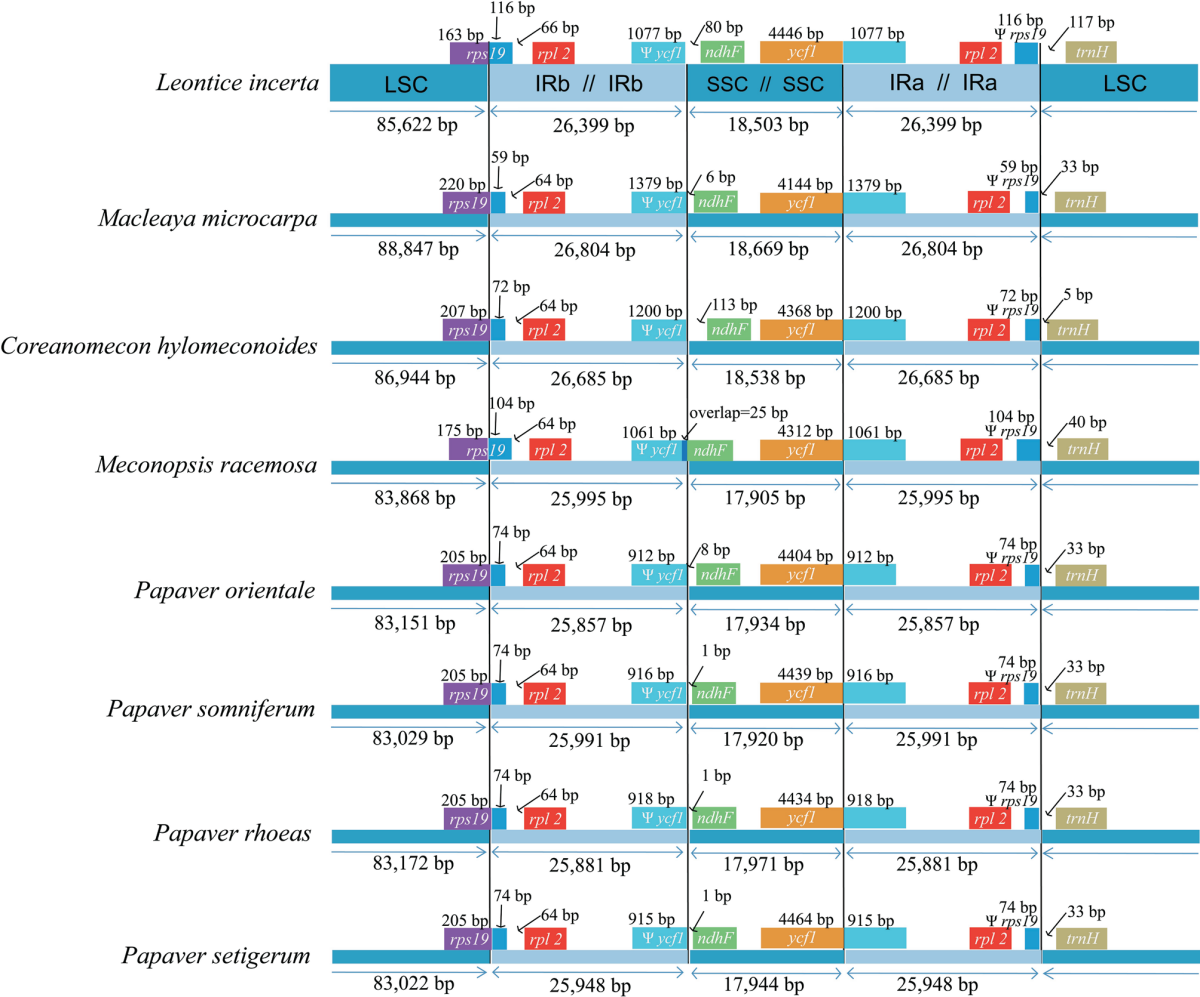
Comparison of the borders of large single-copy (LSC), small single-copy (SSC), and inverted repeat (IR) regions among the seven Papaveraceae chloroplast genomes, with the *Leontice incerta* cp genome shown at the top as the reference.

In terms of the cp genome size observed among the representative Papaveraceae species, the four *Papaver* species were the smallest and had similar genome sizes ranging from 152,799 bp to 152,931 bp (Figure 4). Of the other species, *Macleaya microcarpa* (161,124 bp) exhibited the largest cp genome, while *Meconopsis racemosa* (153,763 bp) had the smallest one.

The *rps*15 gene has been lost from the genomes of *M. racemosa*, *C. hylomeconoides*, *P. orientale*, *P. somniferum*, and *P. setigerum*, although it is present in *P. rhoeas* and the reference genome. In addition, the *ycf*15 gene occurred only in *C. hylomeconoides* compared to the other analyzed cp genomes.

In addition, we compared the exact IR border positions and their adjacent genes between the seven Papaveraceae cp genomes and the reference genome (Figure 4). The results showed that the *ycf*1 gene spanned the SSC/IR_A_ region and the pseudogene fragment of ^y^ *ycf*1 varied from 912 bp to 1379 bp. The *ndh*F gene shares some nucleotides (25 bp) with the *ycf*1 pseudogene in *Meconopsis racemosa* but is separated from ^y^ *ycf*1 by spacers in the other analyzed species. The *trn*H-GUG gene was located in the LSC region of all genomes, but varied from 5 bp to 117 bp apart from the IR_A_/LSC junctions. In addition, the *rps*19 pseudogene appeared in all the representative Papaveraceae species due to the *rps*19 gene extending to the IR region.

### Molecular markers development for *Papaver*

**Figure 5:**
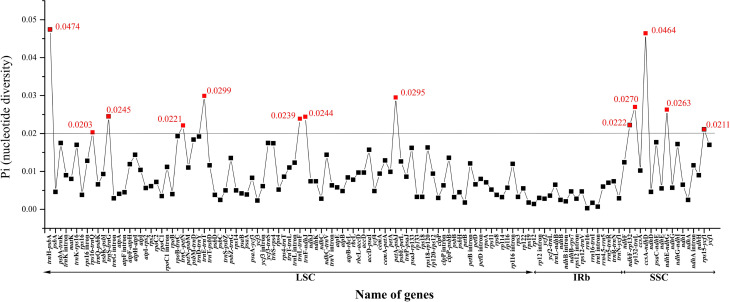
Comparative analysis of the nucleotide variability (Pi) values among the four *Papaver* species.

In order to explore the divergence hotspot regions in *Papaver*, we divided the genome alignment of the four *Papaver* species into non-coding regions, coding genes, and intron regions. We eventually identified 125 loci (53 coding genes, 55 inter-genic spacers, and 17 intron regions) within *Papaver* having more than 200 bp in length (Figure 5). Of these 125 regions, nucleotide variability (*Pi*) values ranged from 0.0003 (*rrn*16) to 0.0474 (*psb*A-*trn*H). Thirteen of these variable loci (*Pi* > 0.02), including *psb*A-*trn*H, *rps*16-*trn*Q, *trn*S-*trn*G, *trn*C-*pet*N, *trn*E-*trn*T, *trn*L-*trn*F, *trn*F-*ndh*J, *pet*A-*psb*J, *ndh*F-*rpl*32, *rpl*32-*trn*L, *ccs*A-*ndh*D, *ndh*E-*ndh*G, and *rps*15-*ycf*1, showed high levels of intrageneric variation. Within the 13 regions, seven varied between *P. setigerum* and *P. somniferum* (Table S2), and these can be candidate markers to identify these two species. Among those markers, *psb*A-*trn*H is the best one to distinguish *P. somniferum* and *P. setigerum*, which are different by seven site mutations.

### Phylogenetic inferences

**Figure 6:**
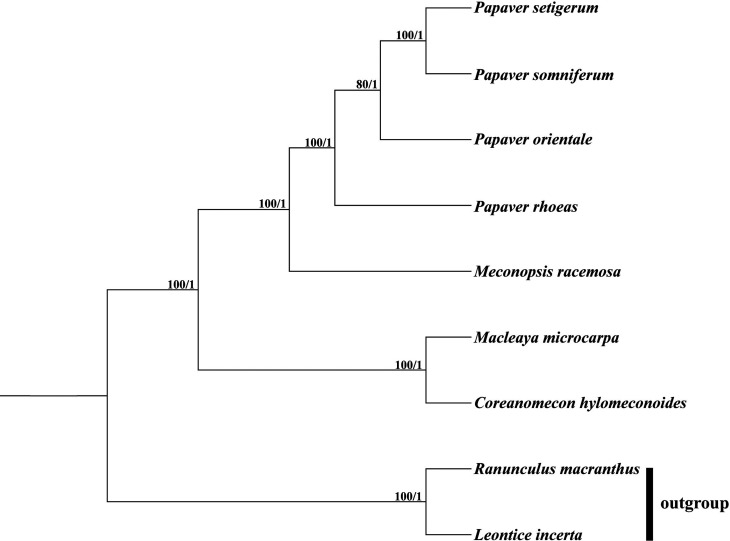
Phylogenetic tree reconstruction of Papaveraceae using maximum likelihood (ML) based on whole chloroplast genome sequences. Numbers above the branches represent bootstrap values from maximum likelihood analyses and posterior probabilities from Bayesian inference, respectively.

The tree topologies from both ML and Bayesian analyses were consistent with each other (Figure 6). All but one node within Papaveraceae have full surport (maximum likelihood bootstrap, MLBS = 100%, Bayesian inference posterior probabilities, BIPP = 1). The four *Papaver* species formed one clade with full support and is sister to *Meconopsis racemosa*. The remaining two species, *Macleaya microcarpa* and *Coreanomecon hylomeconoides*, formed another clade.

## Discussion

In the last decades, the rapid development of high throughput sequencing technologies have greatly reduced sequencing cost. Considering the large number of copies of the plastid genome in a single cell, it is easy to get enough reads to reconstruct a complete cp genome from low-coverage, whole-genome sequencing data (Twyford and Ness, 2017), viz. genome skimming data (Straub *et al.*, 2012). With the publication of many cp genome assembly pipelines (Luo *et al.*, 2012; Jin *et al.*, 2018), cp genome reconstruction by these protocols is more effective than the Sanger method. Since the first complete nucleotide sequence of the cp genome was generated (*Nicotiana tabacum*; Shinozaki *et al.*, 1986), more than 3000 cp genomes have been submitted to GenBank (Jin *et al.*, 2018). In this study, we tried to assemble the cp genome sequence of *Papaver setigerum* using two different pipelines, the CLC Genomics Workbench (CLC Inc., Rarhus, Denmark) and GetOrganelle (Jin *et al.*, 2018). Cp genome sequences produced by the two pipelines were completely identical in terms of both genome size and base information. However, the GetOrganelle pipeline is faster and more effective in assembling a circular cp genomes of *P. setigerum* or other species (we are preparing to publish a comparative study separately), especially for the low coverage data of the whole genome.

In recent years, comparative studies of cp genomes have been applied to a number of focal species (Young *et al.*, 2011), genera (Greiner *et al.*, 2008; Liu *et al.*, 2018b), or plant families (Daniell *et al.*, 2006; Liu *et al.*, 2017). Comparative analyses of cp genomes are useful for phylogenic inference at higher taxonomic levels (Moore *et al.*, 2010; Li *et al.*, 2017), as well as for understanding the evolution of genome size variations, gene and intron losses, and nucleotide substitutions. In the present study, multiple complete cp genomes of representative Papaveraceae species provide an opportunity to compare the sequence variation within the family. No rearrangement, such as translocations and inversions, occurred in gene organization in this family. However, we identified differences in genome size, gene losses, and IR expansion and contraction. The *rps*15 gene is found in most cp genomes in land plants (Tsuji *et al.*, 2007). However, comparative analysis revealed that this gene was found in *P. rhoeas* and the reference genome *Leonitce incerta*, but was not present in other Papaveraceae species. Previous studies have certified that the *rps*15 loss has also appeared in other families (Tsuji *et al.*, 2007; Krause, 2012). Similarly, the function of the *ycf*15 gene has attracted the attention of previous workers (Raubeson *et al.*, 2007; Shi *et al.*, 2013), and it has apparently been lost in other taxa (Liu *et al.*, 2017; Liu *et al.*, 2018). The *ycf*15 gene, which displays a small open reading frame (ORF), is located immediately downstream of the *ycf*2 gene (Dong *et al.*, 2013). In our study, the *ycf*15 gene occurred only in *Coreanomecon hylomeconoides*, located immediately downstream of the *ycf*2 gene but absent in other analyzed cp genomes. These findings suggest that parallel losses of particular genes have occurred during the evolution of land plant cp genomes.

In the genus *Papaver*, almost all of the species are similar in their flower-shapes (two sepals that fall off as the bud opens and four to six petals), colors, and fruits, complicating species identification based on morphological characteristics alone (Osalou *et al.*, 2013; Zhou *et al.*, 2018). Previous studies have identified *Papaver* species using physicochemical methods, including discrete stationary wavelet transform (Zhang *et al.*, 2009), amplified fragment length polymorphism (Lu *et al.*, 2008), as well as phytochemical methods (Osalou *et al.*, 2013). Hosokawa *et al.* (2004) identified *Papaver* species using the plastid gene *rpl*16 and *rpl*16-*rpl*14 spacer sequences. Zhang *et al.* (2015) had verified that *trn*L-*trn*F can be considered a novel DNA barcode to identify the *Papaver* genus, and ITS, *mat*K, *psb*A-*trn*H, and *rbc*L can be used as combined barcodes for identification. Zhou *et al.* (2018) screened five hypervariable regions, including *rpo*B-*trn*C, *trn*D-*trn*T, *pet*A-*psb*J, *psb*E-*pet*L, and *ccs*A-*ndh*D, as specific DNA barcodes. In this study, except for the regions mentioned above, we developed nine additional regions (*rps*16-*trn*Q, *trn*S-*trn*G, *trn*C-*pet*N, *trn*E-*trn*T, *trn*F-*ndh*J, *ndh*F-*rpl*32, *rpl*32-*trn*L, *ndh*E-*ndh*G and *rps*15-*ycf*1) with relatively high levels of intrageneric variation, which can be used for identify *Papaver* species in the future. Moreover, *P. setigerum* was formerly treated as a variety or subspecies of *P. somniferum* due to the similar morphological appearance and chemical signature (La Valva *et al.*, 1985; Osalou *et al.*, 2013). However, the cytological evidence rejects this perspective (Fulton, 1944). Besides, there are seven cp regions varied between *P. setigerum* and *P. somniferum* (Table S2). In the phylogenetic tree of the present study, *P. setigerum* is sister to *P. somniferum* with full support within the *Papaver* clade, which cannot be applied for determining the phylogenetic relationship of these two species due to lack of population sampling. Therefore, more samples for each species in subsequent studies will help us to resolve the genetic relationship between *P. setigerum* and *P. somniferum*.

## Conclusion

In the present study, we assembled the complete chloroplast genome sequence of *Papaver setigerum* based on genome skimming data. The chloroplast genome of *P. setigerum* had a typical quadripartite structure with similar size and organization to other sequenced angiosperms. The evolutionary pattern of cp genomes in Papaveraceae was also detected utilizing seven representative species. Moreover, we screened additional cp hotspots regions for the genus *Papaver*, which will contribute to identification of species in this genus. The inter-genic region *psb*A-*trn*H is the best marker to distinguish *P. somniferum* and *P. setigerum*.

## Supplementary material

The following online material is available for this article:

Table S1 The GenBank accession numbers for other seven species used in comparative chloroplast genome analyses.

Table S2 The variable sites information of seven chloroplast (cp) regions between *P. somniferum* and *P. setigerum*.
