# Supplementary material for: The complete chloroplast genome of Papaver setigerum and comparative analyses in Papaveraceae
Source: Genet Mol Biol. 2020 Aug 17;43(3):e20190272. doi: 10.1590/1678-4685-GMB-2019-0272 (PMC7433754; doi:10.1590/1678-4685-GMB-2019-0272)
Supplement: Supplementary file 1 [file 1415-4757-GMB-43-3-e20190272-s1.pdf]

Genetics and Molecular Biology
https://doi.org/10.1590/1678-4685-GMB-2019-0272

Supplementary Material to "The complete chloroplast genome of *Papaver setigerum* and comparative analyses in Papaveraceae"

Table S1. The GenBank accession numbers for other seven species used in comparative chloroplast genome analyses.

Species	Family	GenBank accession number
Leontice incerta	Berberidaceae	MH940295
Papaver orientale	Papaveraceae	NC_037832
Papaver rhoeas	Papaveraceae	NC_037831
Papaver somniferum	Papaveraceae	NC_029434
Meconopsis racemosa	Papaveraceae	NC_039625
Coreanomecon hylomeconoides	Papaveraceae	NC_031446
Macleaya microcarpa	Papaveraceae	NC_039623
