# Supplementary material for: The complete chloroplast genome of Papaver setigerum and comparative analyses in Papaveraceae
Source: Genet Mol Biol. 2020 Aug 17;43(3):e20190272. doi: 10.1590/1678-4685-GMB-2019-0272 (PMC7433754; doi:10.1590/1678-4685-GMB-2019-0272)
Supplement: Supplementary file 2 [file 1415-4757-GMB-43-3-e20190272-s2.pdf]

Genetics and Molecular Biology
https://doi.org/10.1590/1678-4685-GMB-2019-0272

Supplementary Material to "The complete chloroplast genome of *Papaver setigerum* and comparative analyses in Papaveraceae"

Table S2. The variable sites information of seven chloroplast (cp) regions between *P. somniferum* and *P. setigerum*.

	*psb*A-*trn*H							*trn*L-*trn*F	*trn*E-*trn*T			*rpl*32-*trn*L	*ndh*F-*rpl*32	*rps*16-*trn*Q	*pet*A-*psb*J			
																		1
Nucleotide	0	0	0	0	0	0	2	0	3	3	3	1	3	8	4	4	6	0
position	5	6	6	6	6	6	3	3	2	2	2	3	2	2	0	1	5	7
	9	0	2	3	5	6	4	2	5	6	9	8	2	7	3	9	9	0
P. somniferum	G	A	A	G	A	T	T	C	T	C	A	G	G	C	-	G	-	T
P. setigerum	A	T	C	T	T	C	G	A	-	-	T	T	A	G	G	T	A	C
